# Predictive power of the post-treatment scans after the initial or first two courses of [^177^Lu]-DOTA-TATE

**DOI:** 10.1186/s40658-018-0234-7

**Published:** 2018-12-10

**Authors:** Alexandre Chicheportiche, Simona Grozinsky-Glasberg, David J. Gross, Yodphat Krausz, Asher Salmon, Amichay Meirovitz, Nanette Freedman, Jeremy Godefroy

**Affiliations:** 1 Department of Nuclear Medicine & Biophysics, Hadassah-Hebrew University Medical Center, 91120 Jerusalem, Israel; 2 Neuroendocrine Tumor Unit, Endocrinology and Metabolism Department, Hadassah-Hebrew University Medical Center, 91120 Jerusalem, Israel; 3 Oncology Department and Radiation Therapy Unit, Hadassah-Hebrew University Medical Center, 91120 Jerusalem, Israel

**Keywords:** Peptide receptor radionuclide therapy (PRRT), [^177^Lu]-DOTA-TATE, Neuroendocrine tumors, Kidney dosimetry, Post-treatment scans

## Abstract

**Background:**

The aim of this study was to evaluate the predictive power of the absorbed dose to kidneys after the first course of treatment with [^177^Lu]-DOTA-TATE for neuroendocrine tumors (NETs) on the cumulative kidney absorbed dose after 3 or 4 cycles of treatment. Post-treatment scans (PTS) are acquired after each cycle of peptide receptor radionuclide therapy (PRRT) with [^177^Lu]-DOTA-TATE for personalized radiation dosimetry in order to ensure a cumulative absorbed dose to kidneys under a safety threshold of 25 Gy.

One hundred eighty-seven patients who completed treatment with [^177^Lu]-DOTA-TATE and underwent PTS for dosimetry calculation were included in this retrospective study. The correlation between the cumulative absorbed dose to kidneys after the completion of treatment and the absorbed dose after the first cycle(s) was studied. Multilinear regression analysis was done to predict the cumulative absorbed dose to the kidneys of the subsequent cycles, and an algorithm for the follow up of kidney absorbed dose is proposed.

**Results:**

Patients whose absorbed dose to kidneys after the first cycle of treatment is below 5.6 Gy can receive four cycles of treatment with a cumulative dose less than 25 Gy (*p* < 0.1). For the other patients, the cumulative absorbed dose after 3 or 4 cycles of treatment can be predicted after the second cycle of treatment to allow for an early decision regarding the number of cycles that may be given.

**Conclusions:**

The follow up of kidney absorbed dose after PRRT can be simplified with the algorithm presented in this study, reducing by one-third the number of post-treatment scans and reducing hospitalization time for more than half of the treatment cycles.

## Background

Lutetium-177-DOTA-(Tyr^3^)-octreotate ([^177^Lu]-DOTA-TATE) is used for peptide receptor radionuclide therapy (PRRT) of metastatic progressive neuroendocrine tumors (NETs). Its efficiency has been proven by several studies [1–3] compared to cold somatostatin analogs. Commonly, recommended schedule of treatment with [^177^Lu]-DOTA-TATE consists of a so-called empiric protocol of four fixed doses of 7.4 GBq (200 mCi) infusions every 6–12 weeks [1, 4–6]. This protocol is in accordance with the Food and Drugs Administration (FDA) approval and the European Medicines Agency (EMA) summary of product characteristics [7, 8], provided that the cumulative absorbed dose to the most radiosensitive non-pathological tissues will not exceed safety limits [9–12]. The main late side effects of this treatment are myelotoxicity and renal toxicity [10, 12–16]. Therefore, the limiting factor of the administered dose is the radiation dose to the critical organs, kidneys and bone marrow. Nevertheless, it has been pointed out by Sandström [17] that the dose to bone marrow is rarely a limiting factor (1.5% of the patients). The true threshold of the kidney dose that predisposes patients to toxicity is not precisely known. Preliminary works estimate its value around 23 Gy [17–19] based on external beam radiotherapy, whereas others argue for absorbed doses of about 30 Gy [12, 20]. In our institution, treatment series is stopped if the expected cumulative absorbed dose will exceed 25 Gy, unless otherwise decided by a multidisciplinar staff based on assessment of the individual benefit/risk ratio. The occurrence of toxicity in the patients with high cumulative kidney absorbed dose may be related to risk factors such as diabetes and hypertension [5, 12]. Individual dosimetry by quantitative single photon emission computed tomography (SPECT) after each cycle of treatment monitors the cumulative absorbed dose to the organs at risk to decide whether the patient can receive more cycles of treatment. Following the EANM/MIRD guidelines [21], it is recommended to perform full dosimetry after the first treatment with three SPECT/CT acquisitions at 24, 96, and 168 h after the administration of [^177^Lu]-DOTA-TATE. For the subsequent treatment cycles, only a single SPECT/computed tomography (CT) study approximately 24 h after the treatment is performed assuming an unchanged effective half-life of [^177^Lu]-DOTA-TATE between treatments as proved and proposed by Garske et al. [22].

Based on preliminary observations of the low variability of the kidney absorbed dose across successive cycles of treatment administered empirically, we hypothesized that the cumulative absorbed dose would be correlated to the absorbed dose after the first cycle(s). We therefore retrospectively analyzed the kidney dosimetry data of all the patients who completed serial treatments at our institution, to evaluate the predictive power of the initial or first two courses of treatment on the cumulative absorbed dose to kidneys after completion of treatment.

## Methods

### Patients

Eligibility for PRRT included lesions with high somatostatin receptor (SSTR)-expressing disease on gallium-68-DOTA-(Tyr^3^)-octreotate ([^68^Ga]-DOTA-TATE) positron emission tomography (PET)/CT scan where the tumor uptake was greater than the background liver activity, together with evidence of progressive disease within 12 months, as assessed by combination of increasing biochemical marker (chromogranin A), and new or enlarging lesions on SSTR PET/CT imaging, contrast-enhanced CT, or magnetic resonance imaging (MRI), or symptoms despite conventional management.

Patients were excluded from PRRT if disease demonstrated low SSTR expression, renal impairment (creatinine clearance < 50 ml/min), hypoalbuminemia (< 25 g/L), thrombocytopenia (< 70 × 10^9^/L), pancytopaenia (hemoglobin < 10 g/dL and white cell count < 3 × 10^9^/L), Eastern Cooperative Oncology Group (ECOG) performance score 4, expected survival < 3 months, or confirmed pregnancy.

All consecutive patients who started and completed their series of treatments at our institution between October 23th, 2011 and September 27th, 2017 were included in this study (*n*_p_ = 191 patients). Among these patients, 91 underwent four cycles, 38 three cycles, 39 two cycles, and 23 patients one cycle.

Among the patients who did not complete 4 cycles, 14 were stopped because of an expected kidney absorbed dose after the following cycle higher than 25 Gy. Indeed, at our institution, before giving a subsequent cycle of treatment, the absorbed doses to kidneys during the previous (*p*) treatments are examined and an expected cumulative absorbed dose after the following (*p* + 1) cycle is determined. The latter is calculated as the mean absorbed dose over the previous (*p*) treatments to which is added the cumulative absorbed dose over these (*p*) treatments. We withheld PRRT if the expected cumulated absorbed dose after the subsequent treatment was predicted to exceed 25 Gy for kidneys (except for very specific cases where cost/benefit ratio led us to continue treatment). For instance, a cumulative absorbed dose above 18.75 Gy after the third cycle would lead to stop the treatment.

**Fig. 1:**
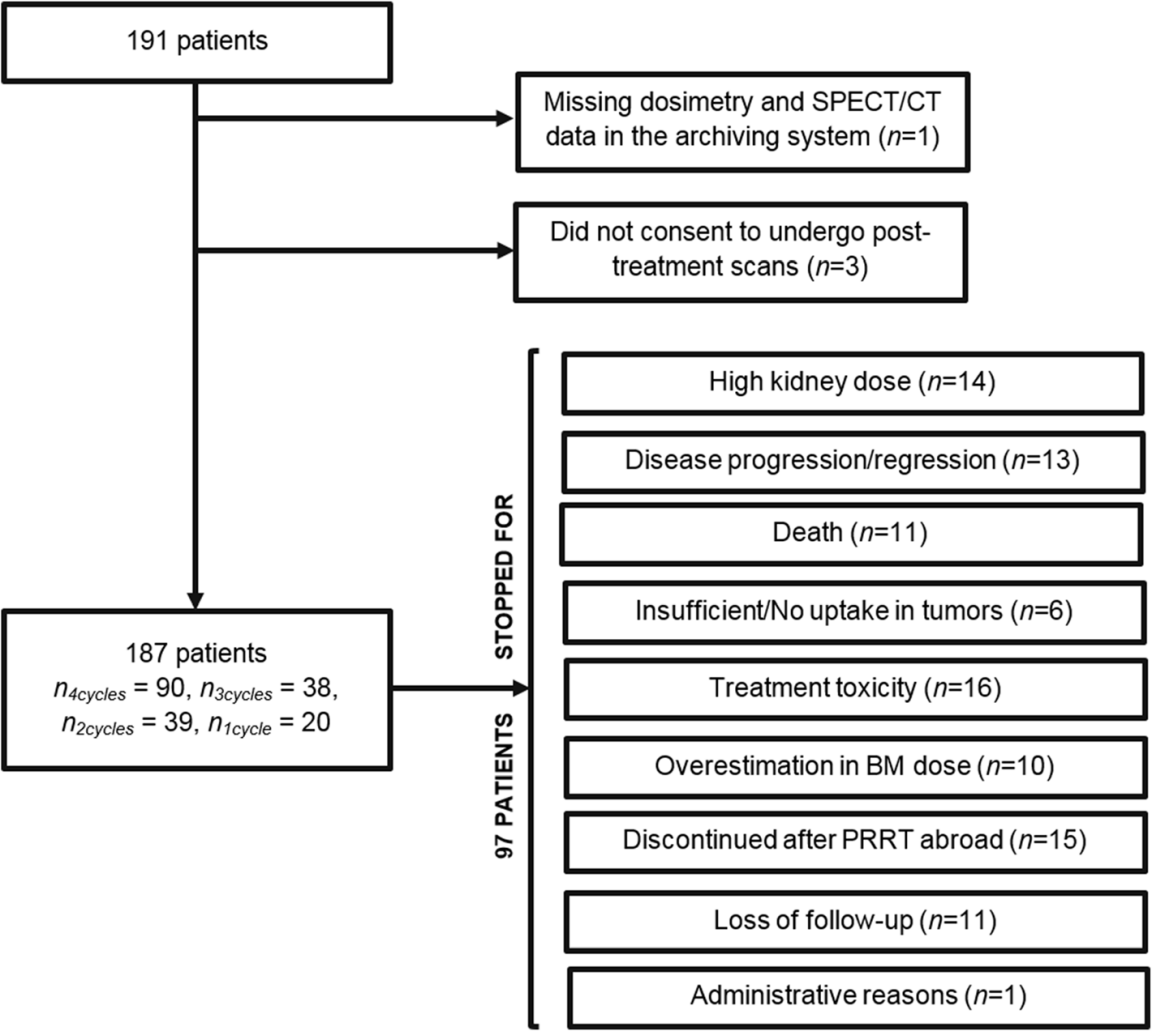
Chart of patient inclusion. Legend: Chart of patient inclusion. *n*_*4*cycles*,*_ *n*_*3*cycles,_ *n*_*2*cycles and_ *n*_*1*cycle_ represent the number of patients included in the study who have completed respectively 4, 3, 2, or 1 cycle(s).

The reasons that led to stop the treatment before the fourth course are shown in Fig. 1. Ten patients (more that 5% of our population of patients) were discontinued due to an excessive bone marrow absorbed dose. As previously mentioned above bone marrow dosimetry is reported in the literature to be rarely the limiting factor, in no more than 1.5% of the patients, a much smaller number than in our institution. This appeared to be the result of an error in the in-house dosimetry calculation software, corrected in January 2017 (after this date, no patient was stopped for this reason). In 15 patients, who had received previous PRRT abroad, treatment at our institution was planned as a short series of only 2–3 “salvage” treatments, so these treatment series were stopped for safety after 1 to 3 cycles regardless to kidney absorbed dose.

In all, 187 patients (110 men, 77 women; average age 58 years, range 11–89 years) were included in this retrospective study. Patient demographics are shown in Table 1.

**Table 1:** Demographic data for the patients included in the study

Characteristic	Value
Total number of patients	187
Age (years)	
Mean ± standard deviation	58 ± 15
Range	11–89
Gender	
Male	110
Female	77
Primary tumor site	
Pancreas	81
Lung	23
Thymus	8
Esophagus	4
Stomach	11
Small intestine	22
Large bowel	9
Pheochromocytoma	5
Paraganglioma	1
Merkle cell carcinoma (skin)	3
Unknown	20
Sites of metastases	
Liver	149
Lymph nodes	95
Bones	65
Lung	15
Peritoneum	15
Pleura	6
Spleen	2
Adrenal	3
Skin or subcutaneous tissue	3
Breast	1
Eye	1

### PRRT therapy

[DOTA0,Tyr3] Octreotate was purchased either from ABX (Radeberg, Germany) or CS Bio Co. (Menlo Park, CA, USA). PerkinElmer, Inc. (Waltham, MA, USA) supplied ^177^LuCl_3_. [^177^Lu]-DOTA-Octreotate was locally prepared by Isorad Ltd. (Soreq NRC, Yavne, Israel). All yields passed a quality control for radiochemical purity including high-performance liquid chromatography (HPLC) and instant thin-layer chromatography (ITLC). Only yields with labeling over 99% were accepted for treatment.

Infusion of amino acids (Vamin 18 g N/L electrolyte-free, Fresenius Kabi) was started at least half an hour prior to administration of the radiopharmaceutical and continued for several hours (4–6 h). As standard protocol, patients received 1.5 L of amino acids solution. The radioactive ligand [^177^Lu]-DOTA-TATE, diluted in 200 ml of saline, was co-administered intravenously over a period of 30 min. For the 187 patients included in this study, the mean activity administered on 581 given cycles was 7.2 ± 0.7 GBq (195.4 ± 18.9 mCi) with a median cumulative activity of 22 GBq (6–31 GBq). The median number of therapy cycles per patient was 3 and the interval between them was 6–12 weeks.

### Post-treatment scan acquisition

All 187 patients included in the study underwent post-treatment scans after each cycle of treatment. Post-treatment scans include a planar whole-body examination under a gamma camera and a SPECT/CT of the abdomen including kidneys, liver, and spleen. Due to organizational constraints in our department, serial SPECT/CTs were acquired 20 h, 25 h, and 7 days after injection of the first therapeutic dose. A single SPECT/CT was acquired after 20 h for the subsequent cycles assuming minor changes in the effective half-life for organs of interest [22]. A post-treatment scan lasts about 35 min (planar acquisition: 15 min; 1 field of view (FOV) SPECT/CT: 20 min).

The first 31 patients underwent imaging on a hybrid SPECT/CT Infinia (International General Electric, General Electric Medical Systems, Haifa, Israel). For all the other patients, images were acquired on a hybrid SPECT/CT Discovery NM/CT 670 camera (International General Electric, General Electric Medical Systems, Haifa, Israel). Both systems combine a dual-head scintillation SPECT camera with an axial FOV of 40 × 54 cm, a NaI(Tl) crystal thickness of 9.5 mm, and 59 photomultiplier tubes (PMT). All functional images were acquired with a 20% energy window around the main photopeak of ^177^Lu (208 keV; 10.4% probability) [23] with medium energy general purpose (MEGP) collimators. Whole body images were acquired with step-and-shoot mode with 180 s per view in a 256 × 1024 matrix. SPECT images were acquired over 360° with 30 angular steps per head and with a 30 s exposure per frame (15-min acquisition) in a 128 × 128 matrix size. Anatomical images were acquired on the Infinia with a four-slice helical CT scanner using a tube voltage of 120 kV and a current of 2.5 mA and with the integrated BrightSpeed multidetector CT (24 rows – maximum 16 slices/rotation) on the Discovery NM/CT 670 using a tube voltage of 120 kV and the Smart current option (80–220 mA).

Before July 2016, calibration of SPECT images was performed on a series of ^177^Lu 20-mL vials placed in the gamma camera FOV with low known activities ranging from 11.1 MBq (0.3 mCi) to about 140 MBq (3.8 mCi). During this time, no scatter correction was applied and thus contributions from scattered photons were ignored.

From July 2016, calibration of SPECT images was based on a series of 30 SPECT acquisitions of a 20-mL vial placed in the center of the gamma camera FOV with a known activity of ^177^Lu ranging from 114.7 MBq (3.1 mCi) to 7215 MBq (195 mCi). The ^177^Lu calibration source was placed in the center of 8 1-L saline bags with two additional ^177^Lu sources in order to simulate an amount of scatter similar to a clinical scan. For scatter estimation, the dual energy window (DEW) method was used. This method consists of measuring the scatter in an energy window juxtaposed just below the main photopeak window (208 keV). Here, the scatter window was placed ± 10% around 166.4 keV as proposed by Beauregard et al. [24]. Then, a pixel-by-pixel correction subtracting the scatter counts from the main photopeak ones is done. This correction uses a weighting factor, which depends on the width of the main peak and scatter energy windows [25]. The measured paralyzing dead-time constant was 0.66 ± 0.04 μs. However, no dead-time correction was applied.

### Image analysis and dosimetry

Image analysis for dosimetry was performed using the General Electric (GE) Dosimetry Toolkit (DTK) software [26] available for the Xeleris 3.0 Workstation (International General Electric, General Electric Medical Systems, Haifa, Israel). The ordered subsets expectation maximization (OSEM) algorithm with attenuation correction (from CT attenuation maps), resolution recovery (for blurring), and scatter correction when available (from July 2016) included in the Xeleris 3.0 workstation were used.

As detailed in a previous work [27], radiation-absorbed doses were computed using an in-house interactive data language (IDL) code developed in our department. The code takes as input data the output file of the GE DTK software and then performs mono-exponential curves fitting, numerical integrations, and dosimetry calculation. The computation of the absorbed dose is based on the method described in [28] for tumors and on the medical internal radiation dose (MIRD) formalism [29] for healthy organs (kidneys, liver, spleen, bone marrow, remainder of the body). Reference [27] provides more precision about the calculation of absorbed dose to bone marrow.

### Statistical methods

Let *D*_i_ be the kidney absorbed dose after the *i*th treatment cycle.

Simple linear regression was used to assess relationship between *D*_1_ and *D*_2_ + *D*_3_ + *D*_4_. We tested the hypothesis that the residuals are normally distributed by a Lilliefors test and that the successive values are mutually independent by a Runs test. Quantitative measures correlation was quantified with the Pearson’s *r* coefficient.

Multivariate linear regression analysis was used to assess the relationships between *D*_3_ or *D*_3_ + *D*_4_ versus *D*_1_ and *D*_2_, and beetween *D*_4_ versus *D*_1_, *D*_2_, and *D*_3_.

We used the predicted *Y* values method [30] (chapter 19 and 20) to predict score on *D*_2_ + *D*_3_ + *D*_4_ for each possible value of *D*_1_, on *D*_3_ or *D*_3_ + *D*_4_ for each possible value of *D*_1_ and *D*_2_ and on *D*_4_ for each possible value of *D*_1_, *D*_2_, and *D*_3_. The probability that the cumulative absorbed dose *D*_T_ after 3 or 4 cycles exceeds 25 Gy was obtained from the *t* statistic values with a *p* value < 0.1 considered significant. For the probability of erroneously precluding a cycle of treatment that would not have led to the cumulative absorbed dose to exceed 25 Gy, a *p* value of 0.05 was considered significant. We deliberately chose a different *p* value for these two probability since the side effects of PRRT are less well established than its efficiency.

Statistical analyses were performed using OriginPro 9.1 software (OriginLab, Northampton, MA, USA) and Matlab (MATLAB and Statistics Toolbox Release 2017a, The MathWorks, Inc., Natick, MA, USA).

## Results

**Fig. 2:**
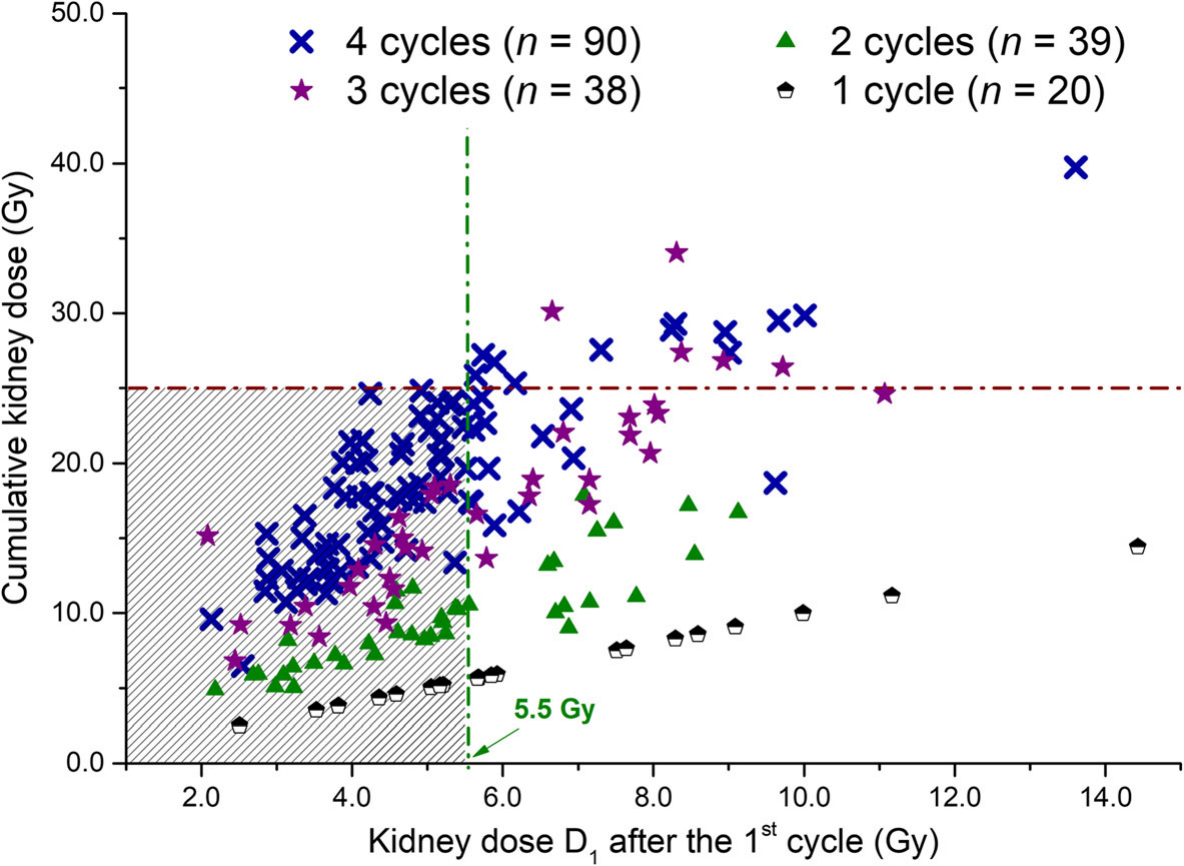
Cumulative kidney absorbed dose versus kidney absorbed dose after the first cycle. Legend: Relationship between the cumulative absorbed dose to kidneys and the absorbed dose after the first cycle. Horizontal dashed line: 25 Gy threshold. Vertical dashed line: threshold under which all patients completed 4 cycles with *D*_T_ under 25 Gy.

Of the 187 patients, 90 completed 4 courses of treatment, of whom 78 had a cumulative kidney absorbed dose *D*_T_ less than the 25 Gy safety threshold. Fourteen patients had their treatment discontinued after the third cycle for kidney dosimetry reasons, since *D*_T_ already exceeded 25 Gy or was expected to exceed this threshold after a fourth cycle. For the patients who did not receive more than 2 cycles, kidney-absorbed dose was never the reason for stopping the treatment. Figure 2 represents the cumulative kidney absorbed dose *D*_T_ for all the 187 patients as a function of *D*_1_.

### Relationship between *D*_1_ and *D*_2_ + *D*_3_ + *D*_4_

**Fig. 3:**
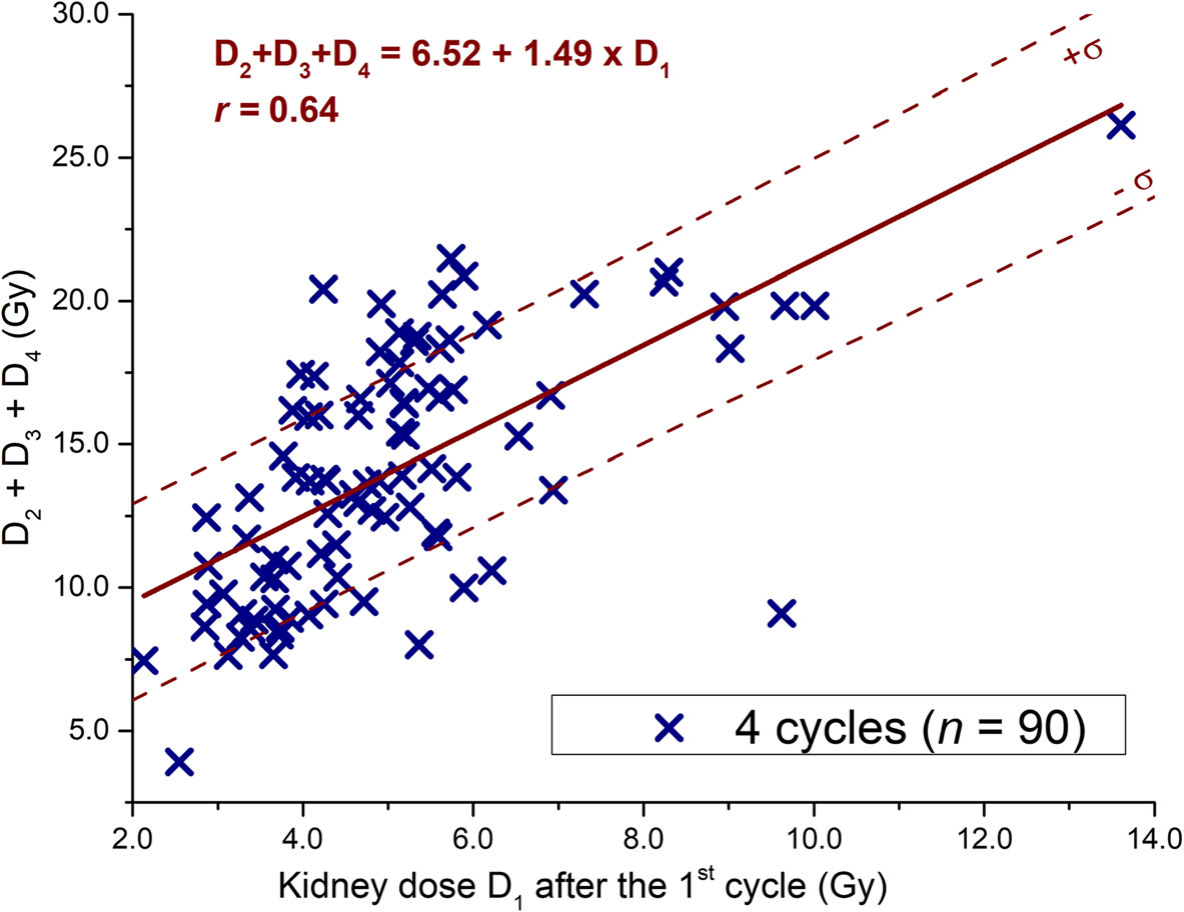
Relationship between *D*_1_ and *D*_2_ + *D*_3_ + *D*_4._ Legend: Relationship between the sum of the doses *D*_2_ + *D*_3_ + *D*_4_ and the dose after the first cycle *D*_1_. The standard deviations of the fitted data are shown in dotted line.

Several functions have been tested to fit the data of the 90 patients who completed 4 cycles. The best fit has been found to be a linear fit, as shown in Fig. 3. The predicted *Y* values method gives a threshold of *T*_1_ = 5.6 Gy such as if *D*_1_ < T_1_, the risk of (*D*_1_ + *D*_2_ + *D*_3_ + *D*_4_) exceeding 25 Gy is less than 10% and less than 5% of exceeding 26.1 Gy. For these values of *D*_1_, it is safe to complete 4 cycles without any additional post-treatment scans (PTS). A value of 5.6 Gy is the 66th percentile of the kidney absorbed dose after the first cycle for our population of patients.

For the other patients in whom treatment was discontinued due to high kidney absorbed dose and with *D*_1_ > 5.6 Gy, we analyzed the incremental amount of information stemming from *D*_2_.

### Relationship between *D*_1_, *D*_2_, and *D*_3_ or *D*_3_ + *D*_4_

**Fig. 4:**
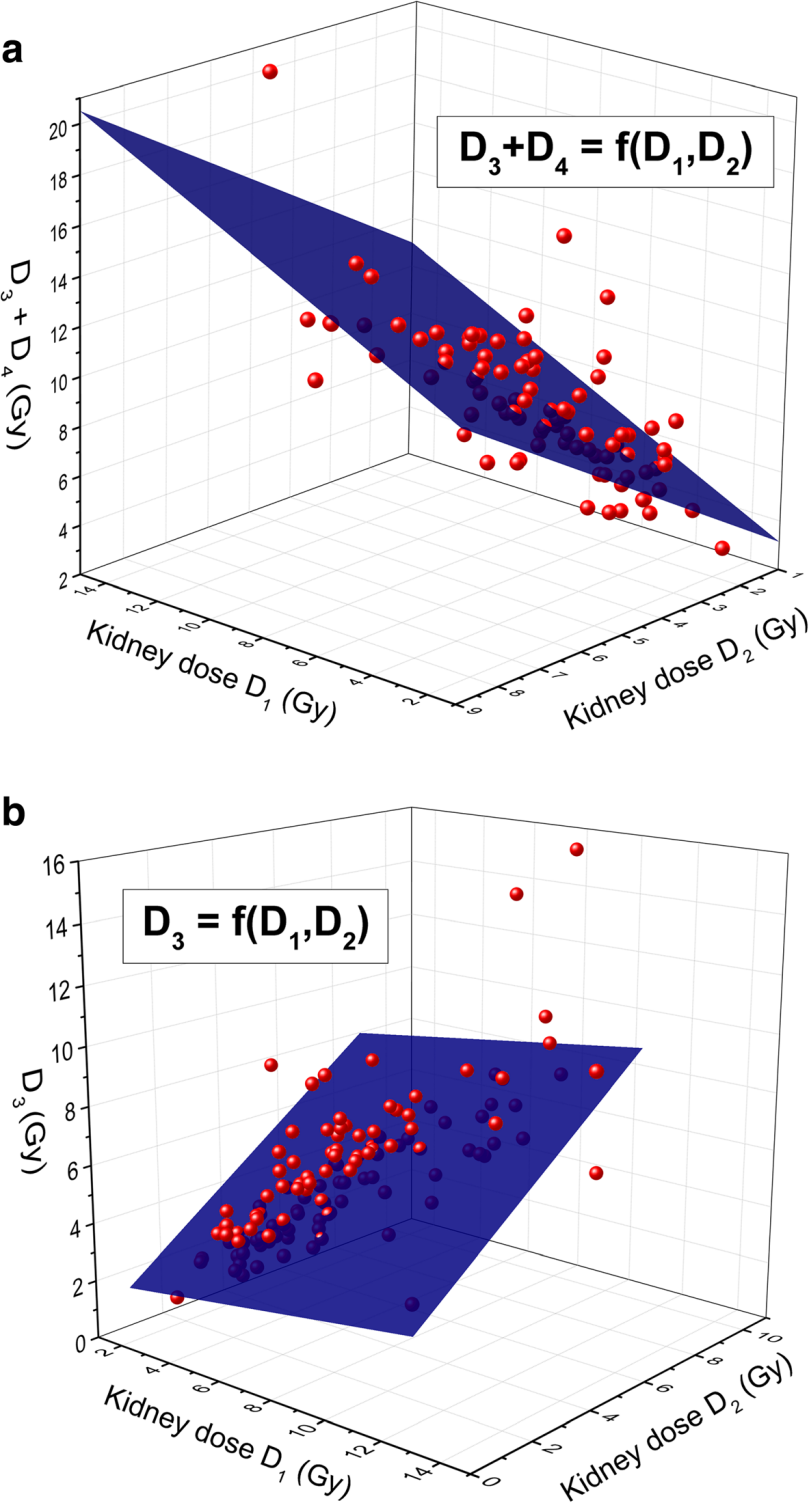
Relationship between *D*_1_, *D*_2_, and *D*_3_ + *D*_4_ or *D*_3._ Legend: Relationship between *D*_1_, *D*_2_ and **a** *D*_3_ + *D*_4_ and **b** *D*_3_. The multilinear fits are shown (blue planes).

For these measurements, a multivariate linear regression analysis was done: for *D*_3_ + *D*_4,_ on the 90 patients who completed 4 courses of treatment, and for *D*_3_ on the 128 patients who completed at least 3 courses. The following relations have been obtained:

1 $$ \left({D}_3+{D}_4\right)={a}_1+{b}_1\times {D}_1+{c}_1\times {D}_2 $$

with *a*_1_ = 1.39, *b*_1_ = 0.56, and *c*_1_ = 1.19 (see Fig. 4a) and,

2 $$ {D}_3={a}_2+{b}_2\times {D}_1+{c}_2\times {D}_2 $$

with *a*_2_ = 0.37, *b*_2_ = 0.14, and *c*_2_ = 0.83 (see Fig. 4b). Expectedly, we find that *c*_1_ > *b*_1_ and *c*_2_ > *b*_2_, reflecting the fact that for treatments that are closer in time more similar dosimetry results are obtained.

**Fig. 5:**
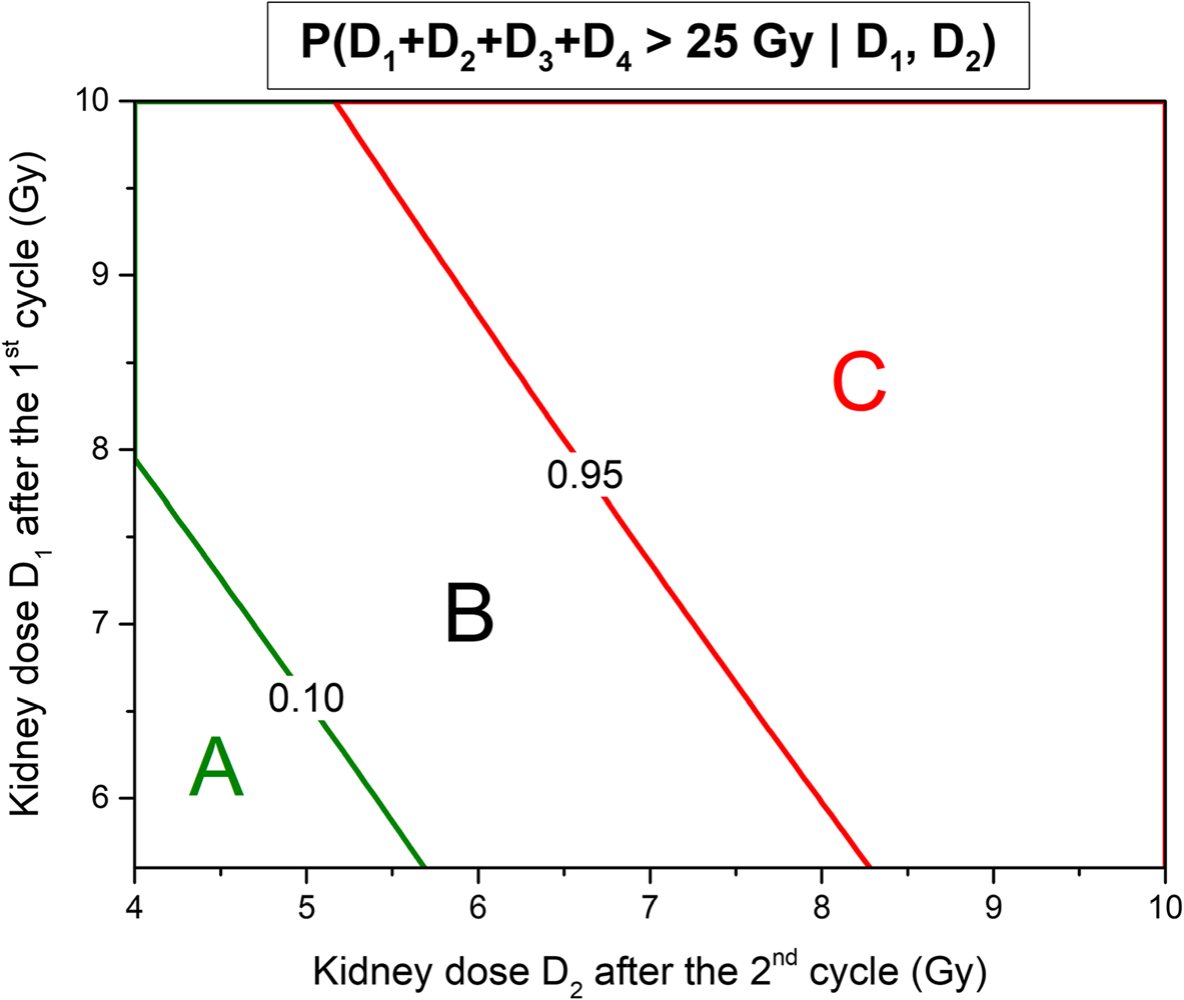
Probability contour plots to exceed 25 Gy after four treatment cycles in function of *D*_1_,*D*_2_. Legend: Probability contour plots of *D*_1_ + *D*_2_ + *D*_3_ + *D*_4_ exceeding 25 Gy as a function of *D*_1_ and *D*_2_. In zone A. the probability is less than 10%. In zone C, it is superior to 95%.

**Fig. 6:**
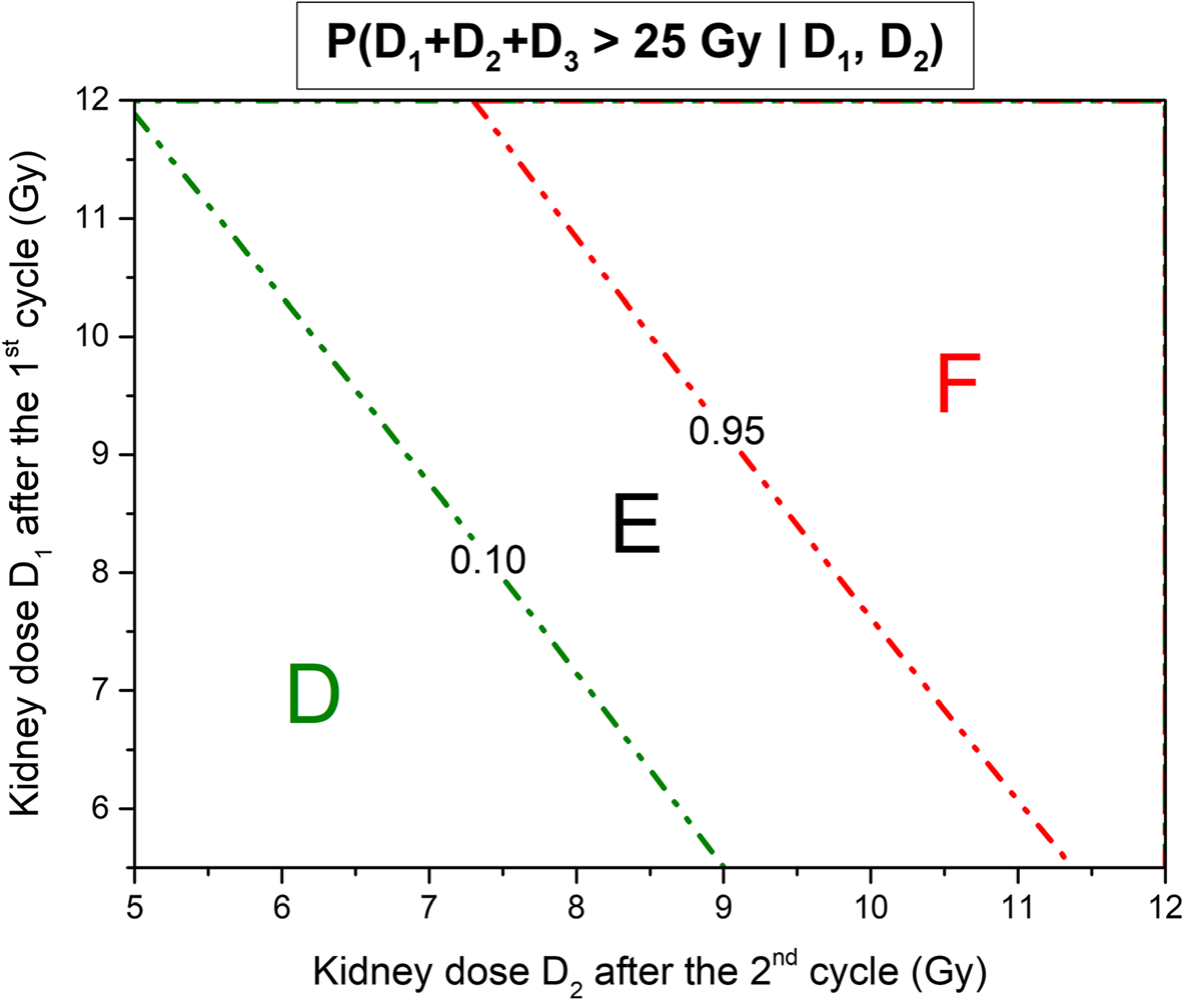
Probability contour plots to exceed 25 Gy after three treatment cycles in function of *D*_1_,*D*_2_. Legend: Probability contour plots of *D*_1_ + *D*_2_ + *D*_3_ exceeding 25 Gy as a function of *D*_1_ and *D*_2_. In zone *D*, the probability is less than 10%. In zone F, it is superior to 95%.

With these models, the risk of (*D*_1_ + *D*_2_ + *D*_3_ + *D*_4_) or (*D*_1_ + *D*_2_ + *D*_3_) exceeding 25 Gy can be computed as a function of *D*_1_ and *D*_2_ using the *Y* predicted values method. Figures 5 and 6 show respectively the probability contour plots to exceed 25 Gy after 4 and 3 cycles as a function of *D*_1_ and *D*_2_. The operational meaning of the preceding results is that for values of *D*_1_ and *D*_2_ in zone A (see Fig. 5), no further PTS is needed to decide to give 4 cycles of treatment (*p* < 0.1). At the other end of the spectrum, for values of *D*_1_ and *D*_2_ at the intersection of zones C and D (see Fig. 6), we can predict the cumulative absorbed dose to be below 25 Gy (*p* < 0.1) after 3 cycles and above after the fourth one (*p* < 0.05). For these values, it is safe and justified to give only 3 cycles of treatment without further follow-up of the kidney dosimetry. In zone E, the decision whether or not administer a third cycle is based on an individual basis, while in zone F the treatment has to be stopped. For absorbed doses in zone B, the information of a third PTS is needed to decide whether to stop after the third treatment.

### Relationship between *D*_4_ and *D*_1_, *D*_2_, *D*_3_

A multivariate linear analysis gives:

3 $$ {D}_4={a}_3+{b}_3\times {D}_1+{c}_3\times {D}_2+{d}_3\times {D}_3 $$

with *a*_3_ = 0.99, *b*_3_ = 0.12, *c*_3_ = 0.19, and *d*_3_ = 0.50. This model lets us predict the expected cumulative absorbed dose after the fourth cycle and to manage the patient accordingly. This approach is more precise than the previous management set up that was to withhold treatment if the cumulative absorbed dose was above 18.75 Gy after the third cycle.

### Management protocol

**Fig. 7:**
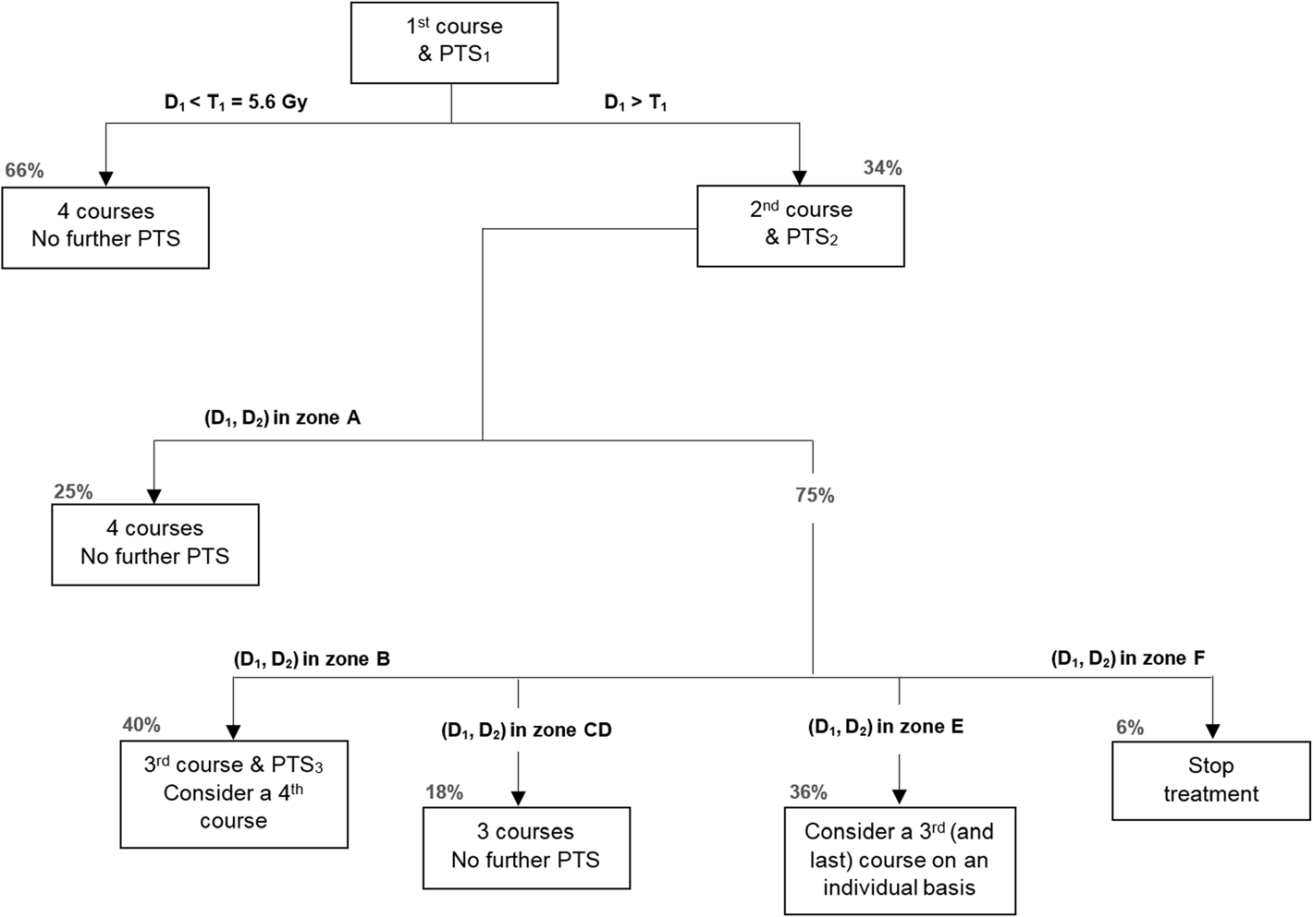
Algorithm for the follow-up of renal dosimetry. Legend: Algorithm for the follow-up of renal dosimetry. Zones A–F are represented in Figs. 5 and 6. Percentages are indicated for our study population.

Based on the observations described in the previous section, we propose an algorithm for the follow-up of renal dosimetry (Fig. 7).

For the 187 patients of the study, using the current empiric protocol, the number of PTS was 946, and the number of inpatient days was 572 (in our institution and with the current protocol patients stay the night following the radiopharmaceutical administration for performing the PTS). Based on the assumption that the future patients treated in our department will be a similar cohort than the population of patients on which our study was done, we project a decrease of about 34% in the number of PTS (from 946 to 626)—a considerable improvement in terms of patient comfort, as well as scanner and technologist time. The number of hospitalization nights is expected to decrease by about 56% (since the patient will not need any more to stay overnight for a dosimetry the day after—for a more thorough overview of the inpatient vs. ambulatory setup issue and patient release, see “Discussion” section).

### Influence of a different threshold

What would be the consequence of a change in the safety threshold of cumulative kidney absorbed dose?

For *D*_1_ = 6.2 Gy, the probability of *D*_T_ exceeding 25 Gy after 4 cycles is 18.7% while by choosing a higher safety threshold of 29 Gy as proposed in refs. [12, 20], this probability would decrease to 2.0%. For patients for whom *D*_1_ < 6.75 Gy, 95% of them would have a cumulative absorbed dose under 29 Gy after 4 cycles.

### Influence of the intra- and inter-observer variability

It has been shown in [27] that the intra- and inter-observer variability for kidney absorbed dose calculation is respectively equal to − 1.0 ± 3.4% and − 6.5 ± 6.8%. Such deviations do not lead to unsafe management of the patients. Indeed, in the worse case scenario where the inter-observer variability reaches 13.3%, if observer 1 calculates an absorbed dose 5.6 Gy and observer 2 a dose of 6.3 Gy (5.6 Gy + 13.3%), the probability to exceed 27 Gy after 4 cycles is inferior to 10%. This is also true for management of the patients using *D*_1_ and *D*_2_, and *D*_1_, *D*_2_, and *D*_3_.

## Discussion

Whether or not the patients treated empirically at fixed activity with [^177^Lu]-DOTA-TATE should have a follow-up of the dosimetry to the critical organs is subject to controversy. In the NETTER-1 trial [3], post-treatment dosimetry was not part of the protocol, although a sub-study on a small sample of patients did include dosimetry. On the other hand, some authors [2, 5, 16, 24, 31] do recommend performing post-treatment scan in order to assess the radiation absorbed dose to critical organs and manage the patients accordingly. Also, there is scarce knowledge about the threshold of maximal safe radiation dose to the kidneys, mainly because of the lack of long-term data for this recently introduced treatment. The current accepted thresholds are inferred from external beam radiation treatments, whose energy deposit is obtained in a fraction of second while being extended over days and weeks in PRRT. Further studies that would prospectively assess the maximum safe absorbed dose to kidneys of [^177^Lu]-DOTA-TATE are needed.

Our study shows that in the scope of four empiric administrations of 7.4 GBq, the dosimetry after each cycle of treatments is often redundant. Indeed, for about two-third of the patients, it is already known after the first cycle that the cumulative dose to kidneys after 4 cycles will not exceed the safety threshold of 25 Gy, thus alleviating the need for further follow up of the kidney dosimetry. What are the logistical implications of these results? In our institution, a large tertiary center with the national referral NET unit treating patients from all over the country and from abroad, the treatment is given in an inpatient setting with patients staying the night following the treatment for radiation safety considerations and for performing the post-treatment scan the next day. Indeed, for dosimetry purpose, PTS cannot be performed too close to the administration of the radiopharmaceutical (about after 24 h as specified by the EANM/MIRD guidelines [21]) in order to model the TACs in their late descending phase [32]. An early measurement in the uptake phase could lead to an over (measurement of high activity in the uptake phase) or underestimation (early measurement in this phase) of the residence times and therefore of the absorbed doses. However, as far as radiation safety is concerned, the outpatient setting is possible as shown by Turner [33] and Calais et al. [34] for patients treated with 7.8 GBq of [^177^Lu]-DOTA-TATE, who attained the radiation exposure release limit 6 h after the injection of the radiopharmaceutical. In Turkey, Abuqbeitah et al. [35] measured a similarly maximum release time of about 5 h after a 5.5 GBq [^177^Lu]-DOTA-TATE therapy. In our center, in the absence of national legislation for patient release after PRRT treatments, our local recommendations are to follow the Australian Radiation Protection and Nuclear Safety Agency where patients can be released if the radiation exposure dose rate at 1 m is below 25 μSv/h [36]. Therefore, for the patients who, based on our results, do not need post-treatment dosimetry, release after a few hours of decay (about 6 h) is possible, without the need to return to the hospital the next day. This would lead to a reduction by 56% of hospitalization nights (notwithstanding a few exceptional cases for whom the clinical presentation or a poor treatment tolerance imposed hospitalization). A 15-min planar whole-body examination could be performed just before the release in order to confirm uptake and visually evaluate the functional response. In the case of progression or of new metastases, a quick SPECT/CT should be performed to explore the findings.

For the calculation of the dosimetry to bone marrow, withholding PTS prevents from estimating the cross-dose. The self-dose, contributing the majority (about 70%) of the bone marrow total dose [17], is estimated from blood. For patients with a good response to treatment, or a stable disease, the PTS after the first cycle can be used to estimate the cross dose. The (very rare) patients that may have a dose to bone marrow near the threshold can have a personalized follow up.

There are several limitations to our study. First, it does not provide a theoretical model that would justify the linear dependence between data from early and late cycles. Second, its validity is limited to the protocol of dosimetry calculation that we use in our institution and its implementation in other centers may need standardization of the camera acquisition parameters and the dosimetry protocol. In others words, it may very well be that the values that we give in our algorithm of follow up may differ in other centers. Most importantly, our study was done in the framework of the empiric protocol of 4 cycles of a fixed activity of 7.4 GBq. It has been shown that patients receiving a fixed amount of activity are not receiving optimal care [37–39], since the dosimetry is not taken into account to optimize the activity injected. There is growing evidence that the injected activity and the number of treatments can be tuned to dosimetry results [40, 41].

The emergence of the idea of personalized dosimetry-based treatments and the fact that the true threshold of toxicity to kidneys may be higher than 25 Gy mean that more than 4 cycles of treatment and/or variable doses higher than 7.4 GBq may be recommended in the future. In this case, the dosimetry would then be fully meaningful and should be recommended after each PRRT cycle.

## Conclusion

In this study, we show how the predictive power of kidney-absorbed dose after the first treatments by [^177^Lu]-DOTA-TATE can be used to reconsider the need for further post-treatment scans for many patients. Our study shows that in the scope of empiric administrations, where 4 cycles of treatments of 7.4 GBq are administered, about one-third of the post-treatment scans for dosimetry are unnecessary. Moreover, according to our local procedures, where patients with a radiation exposure dose rate < 25 μSv/h at 1 m are released after PRRT, this new protocol may reduce hospitalization nights by half. These results can greatly improve the comfort of the patients as well as scanner and technologist time and reduce the treatment expenses without compromising the safety of patient management.

## Abbreviations

[^177^Lu]-DOTA-TATE: Lutetium-177-DOTA-(Tyr^3^)-octreotate
[^68^Ga]-DOTA-TATE: Gallium-68- DOTA-(Tyr^3^)-octreotate
CT: Computed tomography
DEW: Dual energy window
DTK: Dosimetry Toolkit
ECOG: Eastern Cooperative Oncology Group
EMA: European Medicines Agency
FDA: Food and Drugs Administration
FOV: Field of view
GE: General Electric
HPLC: High-performance liquid chromatography
IDL: Interactive data language
ITLC: Instant thin-layer chromatography
MEGP: Medium energy general purpose
MIRD: Medical internal radiation dose
MRI: Magnetic resonance imaging
NETs: Neuroendocrine tumors
OSEM: Ordered subsets expectation maximization
PET: Positron emission tomography
PMT: Photomultiplier tube
PRRT: Peptide receptor radionuclide therapy
PTS: Post-treatment scans
SPECT: Single photon emission computed tomography
SSTR: Somatostatin receptors
